# Interrogating solar photoelectrocatalysis on an exfoliated graphite–BiVO_4_/ZnO composite electrode towards water treatment

**DOI:** 10.1039/c9ra02366f

**Published:** 2019-05-28

**Authors:** Benjamin O. Orimolade, Babatunde A. Koiki, Busisiwe N. Zwane, Gbenga M. Peleyeju, Nonhlangabezo Mabuba, Omotayo A. Arotiba

**Affiliations:** Department of Applied Chemistry, University of Johannesburg South Africa oarotiba@uj.ac.za; Centre for Nanomaterials Science Research, University of Johannesburg South Africa

## Abstract

A novel photoanode consisting of an exfoliated graphite–BiVO_4_/ZnO heterostructured nanocomposite was fabricated. The material was characterised with scanning electron microscopy (SEM), energy dispersive spectrometry (EDS) and X-ray diffraction (XRD). Photoelectrochemical studies were carried out with cyclic/linear sweep voltammetry and chronoamperometry. The solar photoelectrochemical properties of the heterojunction photoanode were investigated through the degradation of rhodamine B in water. The results revealed that the nanoparticles of BiVO_4_ and ZnO were well entrapped within the interlayers of the exfoliated graphite (EG) sheets. Improved charge separation was achieved in the EG–BiVO_4_/ZnO composite electrode which resulted in superior photoelectrochemical performance than individual BiVO_4_ and ZnO electrodes. A higher degradation efficiency of 91% of rhodamine B was recorded using the composite electrode with the application of 10 mA cm^−2^ current density and a solution pH of 7. The highest total organic carbon removal of 74% was also recorded with the EG–BiVO_4_/ZnO. Data from scavenger studies were used to support the proposed mechanism of degradation. The electrode has high stability and reusability and hence lends itself to applications in photoelectrocatalysis, especially in water treatment.

## Abstract 2

Band alignment between ZnO and BiVO_4_ on exfoliated graphite (EG) support.

## Graphical abstract

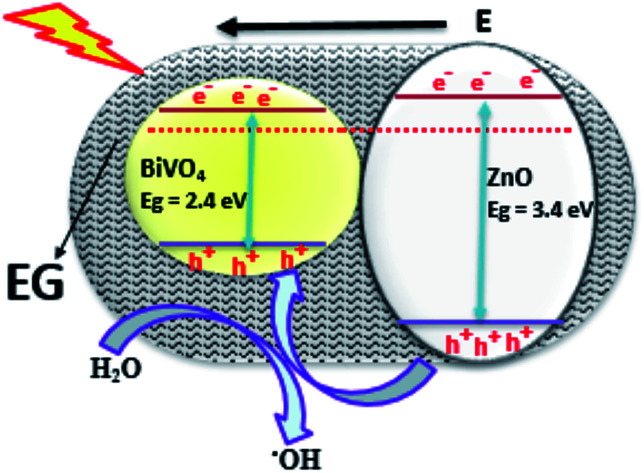

## 1 Introduction

Catalysis, over the years, has been the cornerstone of the improvement and optimisation of many industrial processes including syntheses and production.^1^ The concept of catalysis is also prominent in electrochemical processes where it is generally referred to as electrocatalysis. In electrocatalysis, the rate of an electrochemical reaction occurring on an electrode surface is improved. In fact, many electrochemical processes for energy and sensing^2^ are based on electrocatalysis and photoelectrocatalysis (where light is involved). Recently, catalysis, electrocatalysis and photoelectrocatalysis have found application in the field of water treatment.^3,4^ The challenge of water pollution and incalcitrant pollutants, which are difficult to treat with the conventional water treatment methods, are motivations behind the quests for alternative or complementary approaches to water treatment. For example, the occurrence of dyes, pharmaceuticals and other organic pollutants are on the increase and they have been found to persist in water even after treatment.^5–7^

A promising environmentally friendly technique for a more efficient removal of these dyes is the photoelectrocatalytic (PEC) degradation process.^3^ PEC is a form of advanced oxidation technique which deals with *in situ* production of active oxidizing species such as hydroxyl radicals, superoxide radicals and holes which oxidizes organics to water and carbon dioxide.^4,8^ In PEC, both light and electric energy are used to generate these radicals and it combines both activities of photocatalysis and electrolysis. In other to minimize the cost associated with the use of light energy in generating electron–hole pairs in PEC, solar light is used and the process is referred to as solar photoelectrocatalysis (SPEC).^9^

The electrode commonly used in PEC are often prepared by the immobilization of a photoactive substance which are mostly metal oxide semiconductors on a conducting substrate such as fluorine doped tin oxide glass, anodized titanium sheet, polished stainless steel and conducting quartz glass.^10–12^ Evidently, the performance of PEC process depends largely on the semiconductor used as the anode. Zinc oxide (ZnO) has proven to be a suitable material for PEC applications. ZnO is a non-toxic semiconductor with excellent photocatalytic activities, outstanding chemical stability and high electron mobility.^13^ It has been extensively applied in the removal of organic dyes and pharmaceuticals through both photocatalysis and photoelectrocatalysis.^14–16^ As a result of large band gap energy of ZnO (3.4 eV), its performance is poor with the application of solar energy in PEC process because it best be excited with UV-light (which constitutes less than 5% of the total solar spectrum).^17^ As a result of this drawback, much attention has been given to visible-light photoactive semiconductors such as tungsten trioxide,^18^ bismuth vanadate,^19^ copper(i) oxide,^20^ ferric oxide^21^ and graphitic carbon nitride.^22^ Though these semiconductors perform better than ZnO when visible light is applied in PEC, ZnO is much readily available, easier to synthesize and has higher electron mobility than some of them.^23^ Therefore, ZnO has been combined with visible light active semiconductors to form a heterojunction and such composite has shown higher photocatalytic activity for both degradation of organic pollutants and water spilling for evolution of hydrogen.^23–25^

Basically, an heterojunction is formed when two semiconductors of unequal energy band gaps are combined together in a way that results in band alignment. Several methods such as hydrothermal, chemical bath deposition, calcination, sol–gel process and chemical deposition have been reportedly used for formation of heterojunctions.^26^ The formation of heterojunction has been successfully applied to overcome the problem of rapid recombination of photogenerated electron–hole pairs in visible light semiconductor photocatalysts and to improve their photoactivities.^27^

Bismuth vanadate (BiVO_4_) is a n-type semiconductor with a high visible light photoactivity (absorbs up to 11% of visible light) due to its relatively low band gap energy of 2.4 eV. With the aforementioned properties and its appropriate band edge positions, BiVO_4_ can be a good material for the fabrication of heterojunction with ZnO.^28^ Since the problem of recombination of charge carriers are overcome in BiVO_4_ heterostructured composites, such composites as BiVO_4_/Ag_3_VO_4_,^29^ BiVO_4_/gC_3_N_4_,^30^ Bi_2_S_3_/BiVO_4_ (ref. 31) and BiVO_4_/Cu_2_O^32^ has been applied for degradation of organics and water spilling. Interestingly, BiVO_4_/ZnO heterostructured composites have also been reported to exhibit higher visible light photoactivity as well as lower recombination rate of electron hole pairs suitable for PEC applications. For instance, Feng *et al.*^33^ prepared BiVO_4_/ZnO films on FTO using liquid phase deposition technique in combination with successive ionic layer adsorption and reaction (SILAR) and achieved more than 90% removal efficiency of tetracycline through PEC degradation. Similarly, in the report of Yang and Wu,^34^ photocurrent density of about 3.5 mA cm^−2^ at 1.23 V *vs.* RHE was achieved with the application of BiVO_4_/ZnO in water splitting.

The efficiency of BiVO_4_/ZnO heterostructured composites in PEC applications can further be improved by making use of a conducting substrate that can also minimize the recombination of photogenerated electron–hole pairs by acting as sink for the charge carriers. An example of such substrate is exfoliated graphite (EG), a porous and easily compressible 2D carbon material with high electrical and thermal conductivity. The outstanding electron mobility of EG is essential in channelling photogenerated charge carriers and thereby preventing recombination.^35^ In this study, a thermal treatment technique was used to fabricate BiVO_4_/ZnO heterostructured composite and the composite was combined with exfoliated graphite to form EG–BiVO_4_/ZnO electrode. More importantly, we investigate the solar photoelectrocatalytic behaviours of EG–BiVO_4_/ZnO photoanode using the degradation of rhodamine B dye as a case study. Results on the solar photoelectrochemical responses, degradation efficiency, scavenger study, reusability *etc.* are presented. The positive effect of the inclusion of EG in the heterojunction is confirmed through the enhanced the photoelectrocatalytic degradation or rhodamine B dye at a relatively low photocurrent density.

## 2 Materials and method

### 2.1 Synthesis of ZnO

Hydrothermal technique was employed for the synthesis of ZnO nanoparticles. This was achieved by transferring a mixture containing zinc acetate (0.9041 g, 4 mmol) and 10 mL of PEG400 into a beaker containing 50 mL of absolute ethanol. The mixture was stirred for 15 min and then transferred into a 100 mL Teflon-lined autoclave, which was filled with solid NaOH (0.314 g, 8 mmol). The autoclave was placed in the oven at 140 °C for 24 h, and then allowed to cool to room temperature. The precipitate was collected, washed with absolute ethanol and distilled water thoroughly, and then dried in a vacuum at 60 °C for 6 h.^36^

### 2.2 Synthesis of BiVO_4_

Hydrothermal process was equally employed for the preparation BiVO_4_. Briefly, 1.940 g of bismuth salt and 2.208 g of sodium hydrogen carbonate were dissolved in 70 mL of ethylene glycol to form a solution (A). Another solution (B) containing 0.468 g of Na_3_VO_4_ was dissolved in 10 mL of distilled water and was added to mixture A slowly under stirring. The mixture was further stirred for 30 min and an orange emulsion was formed. The pH of the emulsion was adjusted to 6 and subsequently transferred into a 100 mL Teflon-lined autoclave. The autoclave was sealed and placed in an oven at 180 °C for 24 h. After the reaction duration, the autoclave was allowed to cool to room temperature and the resulting yellow product was collected, washed with ethanol and distilled water several times and then dried at 60 °C for 6 h.^37^

### 2.3 Preparation of BiVO_4_/ZnO composite

A simple thermal treatment method as described by Singh *et al.* was employed in preparing the BiVO_4_/ZnO heterojunction. The synthesized BiVO_4_ and ZnO was taken and mixed in the ratio 1 : 1 and mixed in mortar pestle until a homogeneous composition is obtained. The mixture was then annealed in a programmable furnace at 450 °C for one hour and allowed to cool at room temperature.^38^ Other ratios were also prepared following the same procedure.

### 2.4 Preparation of EG and EG–BiVO_4_/ZnO composite

The exfoliated graphite was prepared through two steps: intercalation and exfoliation. The intercalation was done by soaking natural graphite in concentrated nitric acid and sulphuric acid mixture (ratio of 1 : 3 by volume) for 72 h at room temperature for. The intercalated graphite was washed thoroughly with deionized water to a pH of 7, air-dried. Exfoliation of the intercalated material was done by placing the material in a furnace at a temperature of 800 °C for about a minute.^39^ The EG–BiVO_4_/ZnO composite was prepared by dispersion of the synthesized BiVO_4_/ZnO in absolute ethanol and sonicated for 50 min to obtain uniformity of suspension of the nanoparticles followed by the addition of EG and further sonicated for 30 min (equal masses of EG and BiVO_4_/ZnO were used). The mixture was put in an oven to dry at 100 °C overnight for complete evaporation of the ethanol. EG–BiVO_4_ and EG–ZnO were similarly prepared using BiVO_4_ and ZnO respectively.

### 2.5 Fabrication of EG–ZnO, EG–BiVO_4_ and EG–BiVO_4_/ZnO composite electrodes

The prepared EG–BiVO_4_/ZnO composite was compacted into a pellet (13 mm in diameter) by a hydraulic press at a high pressure of 10 000 psi. The pellet was employed for the construction of an electrode with the use of a copper wire, non-conductive epoxy resin and conductive silver paint. The pellet was placed on the coiled part of the copper wire with the assistance of the conductive silver paint and allowed to dry. The edge of the composite pellet was subsequently sealed with the resin to allow the flow of electricity from only the basal plane. The surface area of the electrode was approximately 1.3 cm^2^. The same procedure was also followed for the fabrication of EG–BiVO_4_ and EG–ZnO electrodes.

### 2.6 Characterisation of prepared materials

The degree of crystallinity, particle size and purity of the prepared photoanodes were determined with the aid of X-ray diffractometer (Rigaku Ultima IV, Japan) using Cu Kα radiation (*k* = 0.15406) with K-beta filter at 30 mA and 40 kV. The surface morphological studies and elemental composition of the materials were carried out on a TESCAN Vega 3 (Czech Republic) scanning electron microscope coupled with energy-dispersive X-ray spectrometer (EDS).

### 2.7 Electrochemical and photoelectrochemical experiments

All electrochemical and photoelectrochemical measurements were conducted on an Autolab 302N (Netherlands) potentiostat/galvanostat with three-electrode configuration. The fabricated electrodes, platinum foil and Ag/AgCl (3.0 M KCl) were employed as working electrodes, counter electrode and reference electrode respectively. A solar simulator (Oriel LCA-100) equipped with a 100 W xenon lamp was used as the light source for PEC experiments. The prepared electrodes were positioned vertically facing the incident light of the simulator and the distance between the photoelectrochemical cell and the light source was kept constant at 10 cm. Photocurrent measurements and linear sweep voltammetry were carried out in a 0.1 M Na_2_SO_4_ solution. Cyclic voltammetry was carried out in a 5 mM solution of [Fe(CN)_6_]^3−/4−^ (prepared in a 0.1 M KCl solution). For the degradation experiments, 50 mL of 10 mg L^−1^ of rhodamine B dye was used with 0.1 M Na_2_SO_4_ solution as supporting electrolyte. Electrochemical degradation experiments were done in the dark. An aliquot of the solution was collected from the reactor at certain time intervals using a disposable syringe. The concentration decay and degradation pattern of the analyte were observed using UV-Visible spectrophotometer (Cary 60, Agilent technologies, Australia) and the total organic carbon removal was recorded using TOC analyser (Teledyne Tekmar TOC fusion). The effects of current density and pH on the removal efficiency were investigated.

## 3 Results and discussion

### 3.1 X-ray diffractometry

**Fig. 1:**
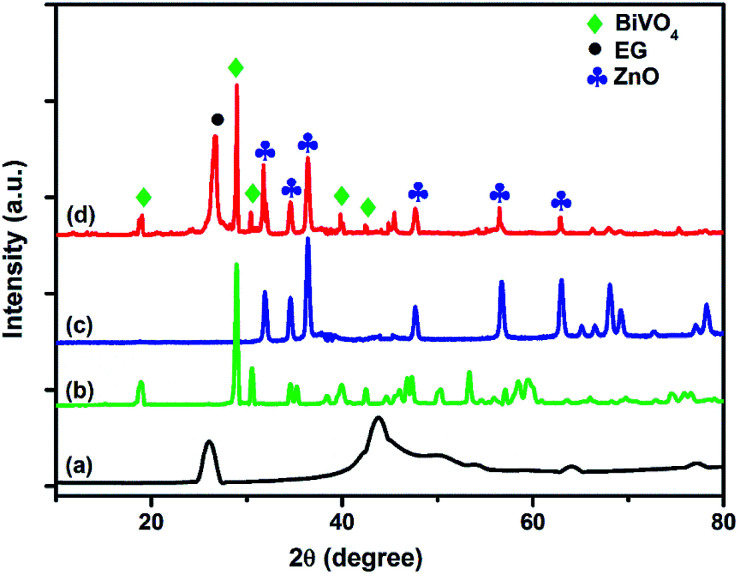
XRD patterns of EG (a), BiVO_4_ (b), ZnO (c) and EG–BiVO_4_/ZnO composite (d).

The XRD pattern of the synthesized BiVO_4_, ZnO, exfoliated graphite (EG) and the EG–BiVO_4_/ZnO composite are shown in Fig. 1. The characteristic diffraction peak of EG was observed at 26.7°. The main peaks observe at 18.8°, 28.9°, 30.4°, 35.1°, 39.7° and 42.1° in the XRD pattern of BiVO_4_ has be indexed as (110, 011), (121), (040), (002), (211) and (150) respectively which correspond to the crystal lattice planes of monoclinic scheelite BiVO_4_ (JCPDS no. 14-0668).^20,40^ The diffraction pattern of ZnO shows peaks at 31.8, 34.5, 36.4, 47.6, 56.7, 63.1, 67.8 and 69.1 which correspond to (100), (002), (101), (102), (110), (103), (112) and (201) respectively to crystalline planes of hexagonal wurtzite ZnO (JPCDS no. 36-1451). All the characteristic peaks of BiVO_4_, ZnO and that of EG can be seen in the XRD pattern of the EG–BiVO_4_/ZnO composite which indicated that the composite was successfully prepared.

### 3.2 Morphological and optical studies of EG, BiVO_4_, ZnO, and EG–BiVO_4_/ZnO

**Fig. 2:**
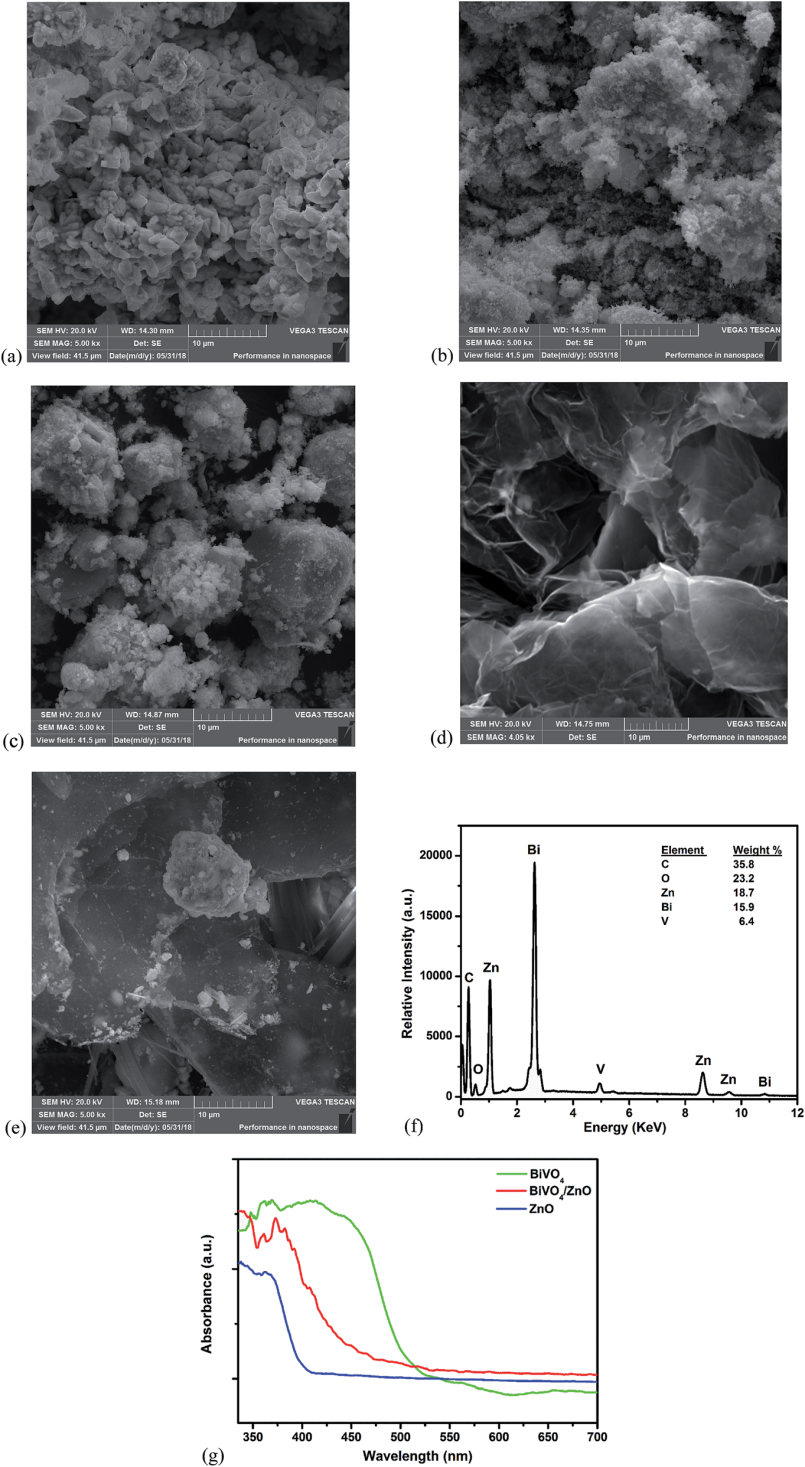
SEM images of BiVO_4_ (a); ZnO (b); BiVO_4_/ZnO (c); EG (d); EG–BiVO_4_/ZnO (e) and EDS spectrum of EG–BiVO_4_/ZnO (f); UV-DRS spectra of BiVO_4_, ZnO and BiVO_4_/ZnO (g).

The surface morphology of the prepared materials was examined using SEM combined with EDS. The SEM image of BiVO_4_ (Fig. 2a) revealed the irregular shapes of BiVO_4_ particles while the ZnO nanoparticles (Fig. 2b) appear as fine agglomerated particles. The BiVO_4_ particles are well integrated between the particles of ZnO with increased agglomeration which revealed the formation of BiVO_4_/ZnO heterostructured composite (Fig. 2c). The graphitic sheets planes of exfoliated can be seen in Fig. 2d with open gravity suitable for the entrapment of particles. The BiVO_4_/ZnO particles were well distributed within the planes of the graphitic sheets of the EG with lesser aggregation within the BiVO_4_/ZnO particles (Fig. 2e). The presence of C, O, Bi, V and Zn elements can be seen the EDS spectrum (Fig. 2f) of the composite material which further confirmed the successful preparation of EG–BiVO_4_/ZnO composite electrode. The UV-Visible diffuse reflectance spectra of the materials confirmed that the synthesized BiVO_4_ and BiVO_4_/ZnO composite absorbs photons in the visible light region and their absorbance edges can be traced to 535 nm and 520 nm respectively (Fig. 2g).

### 3.3 Electrochemical characterisation of electrodes

**Fig. 3:**
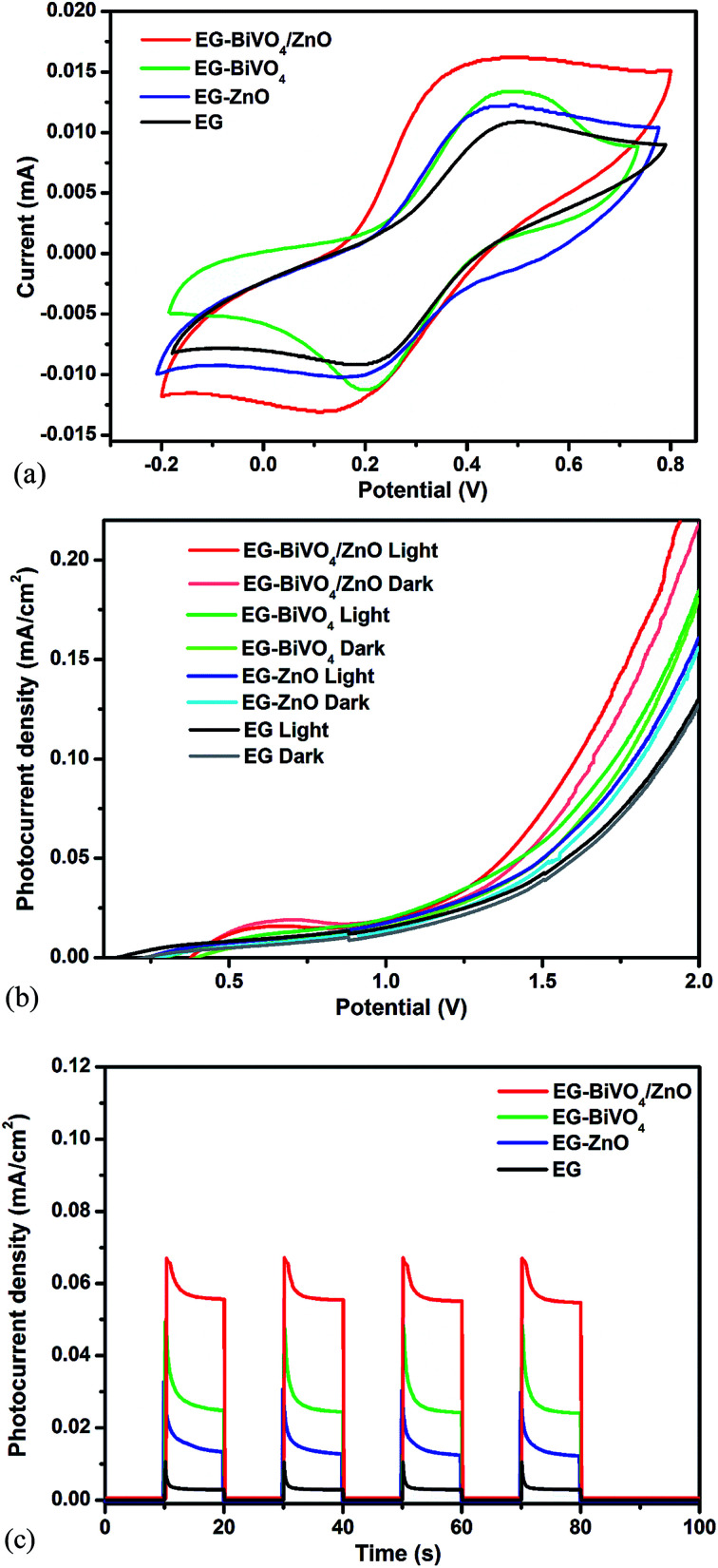
(a) CVs of EG, EG–BiVO_4_, EG–ZnO, EG–BiVO_4_/ZnO composite electrodes using 5 mM [Fe(CN)_6_]^3−/4−^ in 0.1 M KCl solution at a scan rate of 20 mV s^−1^; (b) LSV and (c) photocurrent responses of EG, EG–BiVO_4_, EG–ZnO and EG–BiVO_4_/ZnO in 0.1 M Na_2_SO_4_.

The cyclic voltammograms of EG, EG–ZnO, EG–BiVO_4_ and EG–BiVO_4_/ZnO were recorded at a scan rate 20 mV s^−1^ using 0.1 M KCl solution in 5 mM [Fe(CN)_6_]^3/4^ as the redox probe as shown in Fig. 3a. The EG–BiVO_4_/ZnO electrode showed the highest faradaic peak current than all the other electrodes which suggested that the electrode reaction rate is higher.^41^ Both the EG–ZnO and EG–BiVO_4_ electrode performed better than the bare EG electrode and this could be attributed to that fact that the addition of ZnO and BiVO_4_ to the EG resulted in increased electroactive surface area and thereby increased the conductivity. The electroactive surface area of all the electrodes were calculated using the Randles–Sevcik equation:

1 *i*_p_ = *kn*^3/2^*AD*^1/2^*v*^1/2^*C*

where *A* is the electroactive surface area, *i*_p_ is the peak current, *C* is the redox probe concentration, *D* is the diffusion coefficient (7.6 × 10^−6^ for ferrocyanide), *v* is the scan rate and *k* is a constant (2.69 × 10^5^). The electroactive surface areas obtained are 0.196 mm^2^, 0.239 mm^2^, 0.248 mm^2^ and 0.309 mm^2^ for EG, EG–ZnO, EG–BiVO_4_ and EG–BiVO_4_/ZnO electrodes respectively. The largest surface area of the EG–BiVO_4_/ZnO justified the highest faradaic current obtained with the electrode.

The linear sweep voltammetry of the EG, EG–ZnO, EG–BiVO_4_ and EG–BiVO_4_/ZnO were recorded using 0.1 Na_2_SO_4_ as supporting electrolyte and was carried out both in the dark and light (Fig. 3b). The EG–BiVO_4_/ZnO composite electrode gave the highest current as compared to the other electrodes both in the dark and in the light. The enhanced current observed is due to the formation of heterojunction between the BiVO_4_ and ZnO which resulted in better separation of the electron–hole pairs. This was further confirmed by the photocurrent response with the application of 1.5 V bias potential (Fig. 3c). The EG–BiVO_4_ electrode gave a higher photocurrent (0.049 mA cm^−2^) than that of EG–ZnO (0.031 mA cm^−2^) and this is due to the fact that visible light was used to irradiate the electrode and BiVO_4_ has higher photoactivity than ZnO in the visible light region due its lower band gap. Overall the EG–BiVO_4_/ZnO electrode gave the best photocurrent response of 0.069 mA cm^−2^ and this confirmed that the heterojunction formed between the BiVO_4_ and ZnO resulted in increased interfacial charge transfer and lowered the recombination rate of the charge carriers. Moreover, it is quite interesting to observe that the BiVO_4_, ZnO and BiVO_4_/ZnO electrodes with EG as the conducting substrate gave higher photocurrent responses than their counterpart electrodes with FTO used as the conducting substrate previously reported.^33,34^ This is mostly due to the fact that EG also enhanced effective charge separation by acting as a reservoir for the photogenerated charge carriers. In addition to this, carbon materials can also serve as dopants for semiconductors generating internal electric field contributing to formation of effective heterojunction.^42–44^

### 3.4 Degradation of rhodamine B

**Fig. 4:**
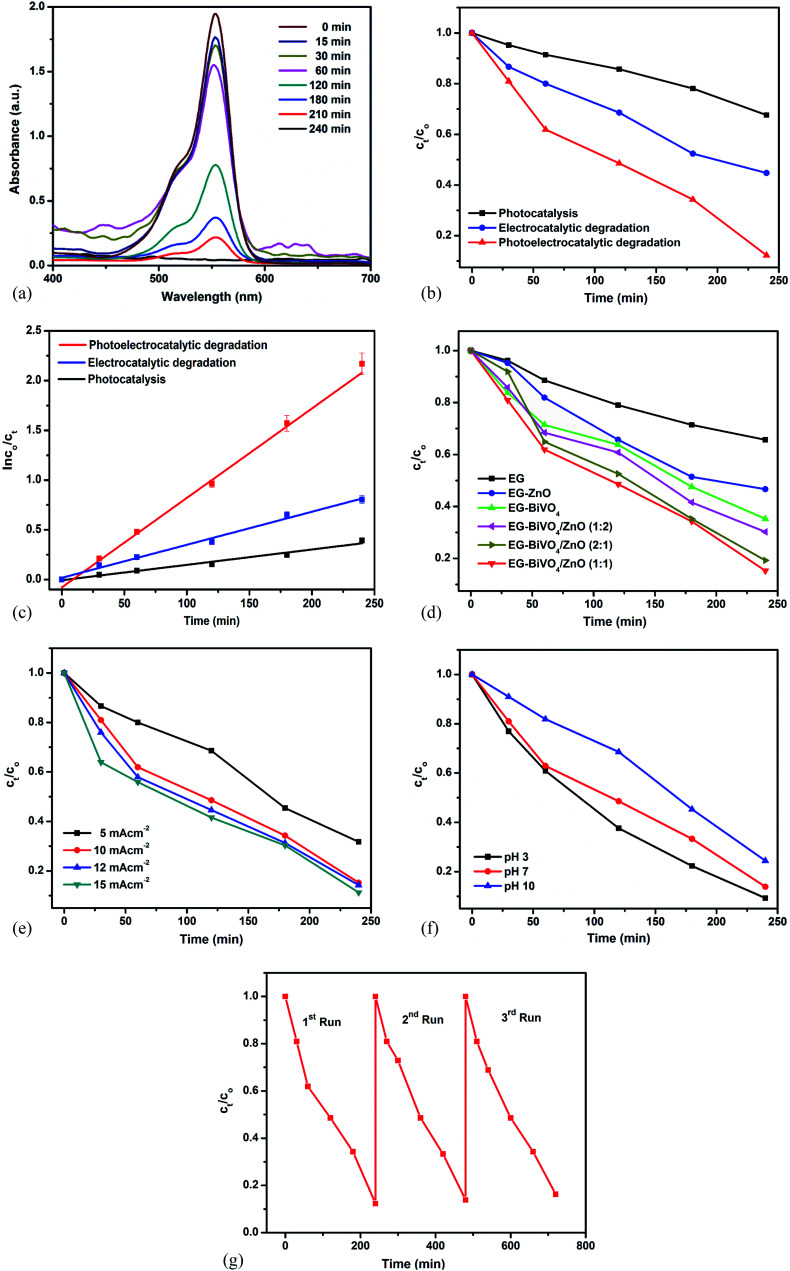
UV-Vis spectra of PEC degradation of rhodamine B (a); normalised concentration decay *versus* time plot for photocatalytic, electrocatalytic and photoelectrocatalytic degradation of rhodamine B dye on EG–BiVO_4_/ZnO electrode (b) and corresponding kinetics plots (c); normalised concentration decay *versus* time plot for PEC degradation of rhodamine B dye on EG–ZnO, EG–BiVO_4_, EG–BiVO_4_/ZnO electrodes (d); effects of current density (e) and pH (f) on degradation of rhodamine B dye; cycle experiments for the degradation of rhodamine B dye on EG–BiVO_4_/ZnO electrode (g).

The prepared electrodes were applied in the solar photoelectrocatalytic (PEC) degradation of rhodamine B dye with initial concentration of 10 mg L^−1^. The degradation efficiency was monitored using UV-Visible spectrophotometer at a wavelength of 554 nm. As shown in Fig. 4a, there was decrease in the intensity of the peak at 554 nm with increase in the reaction time revealing the reduction in the concentration of the dye. Degradation efficiency of 91% was achieved using the EG–BiVO_4_/ZnO composite electrode in the photoelectrocatalytic degradation process. When the composite electrode was applied in electrochemical (EC) degradation (without the application of solar light), the degradation efficiency dropped to 56% while in photocatalysis (PC), 37% degradation efficiency was recorded (Fig. 4b). The total organic carbon (TOC) removal obtained were 74%, 46% and 35% using PEC, EC and PC respectively. This observation revealed that application of biased potential plays a vital role in the degradation process. Additionally, when the electrode is irradiated with the solar light, the electrode absorbs photons and electron–hole pairs are generated within the BiVO_4_ and ZnO. The holes act as oxidants for breaking down the dye molecules and also react with water to generate hydroxyl radicals which are stronger oxidants necessary for the rapid oxidation of the dye molecules. The combined effect of electrical energy and solar energy accounted for the highest degradation efficiency achieved in the PEC process. The degradation rate constants for the three processes (PC, EC and PEC) were obtained by fitting the data into Langmuir–Hinshelwood pseudo first order kinetic model equation. As shown in Fig. 4c, the rate of the PEC process was the fastest with apparent rate constant of 0.00921 min^−1^ when compared to both PC and EC processes with rate constants of 0.00154 min^−1^ and 0.00332 min^−1^ respectively.

Among all the electrodes prepared, the bared EG electrode performed least with 27% degradation efficiency. The EG–BiVO_4_ electrode performed better (61% efficiency) than the EG–ZnO (52%) electrode because the BiVO_4_ has higher photocatalytic activity in the visible light region due to its lower band gap (Fig. 4d). The formation of BiVO_4_/ZnO heterojunction was deduced through the improved photoelectrocatalytic activity of all the composite electrodes with different ratios of BiVO_4_ to ZnO. The formation of heterojunction enhanced better charge separation and lowered the rapid recombination rate of electron–hole pairs resulting in better light harvesting. The EG–BiVO_4_/ZnO consisting of BiVO_4_ and ZnO in ratio 1 : 1 gave the highest degradation efficiency and was therefore used for all other studies.

The effects of parameters such as current densities and solution pH on the photoelectrocatalytic process were as studied. The degradation efficiency increased with increased in the applied current densities from 5 mA cm^−2^ to 15 mA cm^−2^ (Fig. 4e). The increase from 5 mA cm^−2^ to 10 mA cm^−2^ (62% to 91%) was more pronounced as compared to the increase observed from 10 mA cm^−2^ to 15 mA cm^−2^ (91% to 95%). This revealed the dependence of hydroxyl radicals production on the applied current densities but when too high current densities are applied, it could result in oxygen evolution which hinder the generation of hydroxyl radical and thereby limit the degradation efficiency.^45^ More so, the application of higher current densities is not cost and energy effective. Increase in pH from 3 to 10 (Fig. 4f) resulted in decrease in the degradation efficiency which confirmed the suitability of acidic medium for degradation of rhodamine B dye.^46^

An ideal photoanode for degradation of organics is expected to be stable and reusable. The stability and reusability of the prepared EG–BiVO_4_/ZnO in PEC degradation process was also investigated by carrying out repeating experiments (cycles) using the same electrode. The electrode was cleaned with deionized water after each cycle. As shown in Fig. 4g, the degradation efficiency dropped by less than 1% after the third cycle which implies that the electrode is relative stable and can be reused for PEC degradation.

### 3.5 Proposed mechanism of degradation and scavenger studies

In PEC processes, photogenerated holes, superoxide and hydroxyl radicals play active role in oxidizing the organic compounds. The formation and reactions of these reactive species with rhodamine B molecules are given in eqn (2)–(7).

2 *hv* + BiVO_4_/ZnO → BiVO_4_/ZnO (h_VB_^+^ + e_CB_^−^)

3 e_CB_^−^ + O_2_ → ·O_2_^−^

4 h_VB_^+^ + H_2_O → ·OH + H^+^

5 ·OH + rhodamine B → CO_2_ + H_2_O

6 ·O_2_^−^ + rhodamine B → CO_2_ + H_2_O

7 h_VB_^+^ + rhodamine B → CO_2_ + H_2_O

**Fig. 5:**
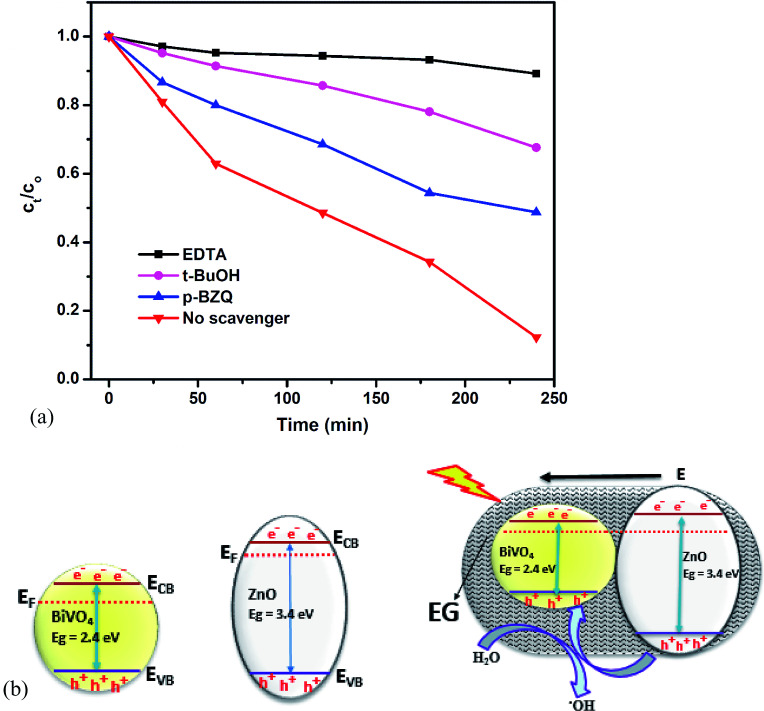
Scavenger studies of the PEC degradation of rhodamine B dye on EG–BiVO_4_/ZnO (a); band alignment between ZnO and BiVO_4_ (b).

To get a clearer picture of the roles of the active species in the PEC degradation of rhodamine B, scavenger study was carried out. This was done by using ethylenediaminetetraacetate salt (EDTA), *t*-butanol (*t*-BuOH) and *p*-benzoquinone (*p*-BZQ) to suppress the effect of holes, hydroxyl radicals and superoxide radicals respectively.^47,48^ The results are shown in Fig. 5a. Upon the addition of EDTA, the degradation efficiency dropped drastically to less than 10% which revealed that the photogenerated holes play a major role in the degradation of the rhodamine B dye. With the addition of *t*-BuOH and *p*-BZQ the degradation efficiencies were 31% and 52% respectively suggesting that both hydroxyl radicals and superoxide radicals play a lesser role in the degradation of the dye. The pronounced role of the photogenerated holes in degradation of the dye molecules could be attributed to a better separation of the electron–hole pairs in the BiVO_4_/ZnO heterojunction making more holes available to oxidize the dye molecules. This observation provided a glimpse into the band alignment between ZnO and BiVO_4_. Considering the band edge positions of intrinsic BiVO_4_ and ZnO, a type I heterojunction can be proposed in which the charge carriers accumulate onto the BiVO_4_ and this will not result in proper charge separation as the charge carriers can still recombine in the surface of the BiVO_4_. This was unlikely to happen since carbons (from EG) act as dopants for both ZnO and BiVO_4_ leading to non-negligible internal electric field. Therefore, Fermi energy levels (*E*_F_) of the two semiconductors aligned through the movement of electrons from the ZnO (higher *E*_F_) to BiVO_4_ (lower *E*_F_) and the direction of internal electric field (*E*) was from ZnO to BiVO_4_ preventing the movement of electrons in the same direction after the formation of heterojunction. On the other hand the direction of the internal electric field favored the movement of holes in the same direction and hence holes were most efficient separated onto the valence band of BiVO_4_ and available for oxidizing the rhodamine B molecules.^42^ This deduction is illustrated in Fig. 5b.

## 4 Conclusion

In this work, we have prepared a novel heterojunction photoanode of EG–BiVO_4_/ZnO. The formation of BiVO_4_/ZnO heterojunction with enhanced separation of charge carriers was confirmed through the photocurrent response of the EG–BiVO_4_/ZnO composite electrode which was higher than that of both the EG–BiVO_4_ and EG–ZnO. The exfoliated graphite, used as the conducting substrate, also improved charge separations by acting as a sink for the charge carriers. When applied for the photoelectrocatalytic degradation of rhodamine B, a degradation efficiency of 91% (from visible spectroscopy) and a total organic carbon removal of 74% were recorded with the EG–BiVO_4_/ZnO heterostructured photoanode. Scavenger studies revealed that photogenerated holes played a major role in the degradation of the dye since the band alignment between the BiVO_4_ and ZnO resulted in better charge separation of the holes. The composite electrode has high stability and the reaction rate is fast with apparent rate constant of 0.00921 min^−1^. The electrode has a great potential for PEC applications in wastewater treatment for the removal of organics.

## Conflicts of interest

There are no conflicts to declare.

## Supplementary Material
